# Design of different strategies of multivalent DNA-based vaccination against rabies and canine distemper in mice and dogs

**DOI:** 10.1186/1743-422X-9-319

**Published:** 2012-12-27

**Authors:** Leila Touihri, Sami Belhaj Ahmed, Yacine Chtourou, Rahma Daoud, Chokri Bahloul

**Affiliations:** 1 Institut Pasteur de Tunis, MMVDB, 13, Place Pasteur BP-74, Tunis-Belvedere, 1002, Tunisia

**Keywords:** Rabies, CDV, DNA Vaccine, Multivalent, Public health, Zoonose

## Abstract

**Background:**

During the vaccination campaigns, puppies younger than 3 months old are not targeted and remain unvaccinated for at least the first year of their lives. Almost half of the reported rabid dogs are 6 months or younger. Hence, we should recommend the vaccination against rabies of young puppies. Unfortunately, owing to the exposure of puppies to infections with either canine parvovirus (CPV) or distemper virus (CDV) after the intervention of the vaccinators, owners are reluctant to vaccinate puppies against rabies. Therefore, it is necessary to include the CPV and CDV valences in the vaccine against rabies. Multivalent DNA-based vaccination in dogs, including rabies and distemper valences, could help in raising vaccine coverage.

**Methods:**

We have designed monovalent and multivalent DNA-based vaccine candidates for *in vitro* and *in vivo* assays. These plasmids encode to the rabies virus glycoprotein and/or the canine distemper virus hemagglutinin. The first strategy of multivalent DNA-based vaccination is by mixing plasmids encoding to a single antigen each. The second is by simply fusing the genes of the antigens together. The third is by adding the foot and mouth disease virus (FMDV) 2A oligopeptide gene into the antigen genes. The last strategy is by the design and use of a bicistronic plasmid with an “Internal Ribosome Entry Site” (IRES) domain.

**Results:**

The monovalent construct against canine distemper was efficiently validated by inducing higher humoral immune responses compared to cell-culture-derived vaccine both in mice and dogs. All multivalent plasmids efficiently expressed both valences after *in vitro* transfection of BHK-21 cells. In BALB/c mice, the bicistronic IRES-dependant construct was the most efficient inducer of virus-neutralizing antibodies against both valences. It was able to induce better humoral immune responses compared to the administration of either cell-culture-derived vaccines or monovalent plasmids. The FMDV 2A was also efficient in the design of multivalent plasmids.

**Conclusions:**

In a single shot, the design of efficient multivalent plasmids will be very beneficial for DNA-based vaccination against numerous diseases.

## Background

Rabies is a major public health concern and dogs represent the main vector and reservoir, especially in developing countries [1]. Around the world, the rabies burden is estimated each year at around 60,000 human deaths and more than 10 million cases of post-exposure prophylaxis [2]. It is largely accepted that any attempt to completely control canine rabies has to be through a strategy of mass vaccination of dogs [3]. Such vaccination campaigns must reach at least 70% of vaccine coverage. Several successful programs have been conducted in South America [4,5] and in North Africa [6]. However, even though these programs have resulted in the control of enzootic dog rabies in many urban regions, they have not been effective in eliminating the disease in most of these countries [7,8].

Previously, we have shown that mass vaccination of dogs against rabies in Tunisia has yielded rather suboptimal outcomes [9]. These can be attributable to low vaccination coverage rates; in many parts of the country, the vaccination coverage can be very low, which allows the maintenance of a reservoir of rabies. In addition, in the same work, we have recommended the vaccination of newborn puppies in order to raise the vaccine coverage rates. Unfortunately, puppy owners tend to be reluctant to vaccinate them against rabies because they can be exposed intempestively to infections of either canine parvovirus (CPV) or canine distemper virus (CDV) through the intervention of the vaccinators. Consequently, we think that it is necessary to include other valences such as CPV and CDV in the inoculated vaccine (in addition to the rabies valence).

DNA-based vaccinations against rabies and CDV have been largely and efficiently investigated in different animal models [10,11]. Previously, we have constructed the plasmid pCMV3ISS-GPV which encodes to the glycoprotein of the rabies PV strain [12]. We have shown that a rabies post-exposure vaccination in BALB/c mice, based on a single administration of pCMV3ISS-GPV, is at least as effective as five-injections of cell-culture-derived vaccine. Furthermore, we have shown that the use of DNA-based vaccination in dogs with pCMV3ISS-GPV is more efficient than after the use of the best commercially available cell-culture-derived vaccine, under experimental as well as field conditions [13].

Different strategies of multivalent DNA-based vaccination have been reported. Hence, we can design a bicistronic DNA plasmid with an internal ribosome entry site (IRES); or we can construct a plasmid encoding to a fusion poly-protein, with or without an internal cleavage factor, such as including the foot and mouth disease virus (FMDV) oligopeptide 2A.

More then two decades ago, an alternative mechanism of translation and initiation by direct ribosome binding to the IRES domain of picornaviruses and encephalomyocarditis virus was reported [14,15]. Other classes of RNA viruses (Hepatitis C Virus), or DNA viruses [16], or even a subset of eukaryotic cells during conditions of distress [17], are capable of following the IRES pathway. The IRES can be used to engineer multicistronic vectors, which may express several open frames from the same transcript. However, it has been reported that cap-dependent translation is more efficient than IRES translation [18,19].

The large sizes of the IRES domains (~0.5 kb) and the difficulties of ensuring a well-balanced co-expression of multiple genes have promoted an attractive alternative based on the FMDV oligopeptides 2A [20]. FMDV 2A are relatively short peptides of 20 amino acids containing the consensus motif Asp-Val/Ile-Glu-X-Asn-Pro-Gly-Pro. During translation, the 2A interacts with the exit tunnel of the ribosome to induce the “skipping” of the last peptide bond at the C-terminus of 2A [21]. Then the ribosome continues the translation downstream along the gene, after releasing the first protein fused in the C-terminus of 2A. It was already reported that the 2A peptide activities are functional in a wide variety of eukaryotic cells derived from yeasts, plants, and insects to mammals [22].

In order to set up a multivalent DNA-based vaccine against rabies and canine distemper we have compared numerous approaches in this preliminary assessment. The first approach is by using a mixture of plasmids encoding to a single antigen each; the second approach is by constructing a plasmid which encodes to a fusion of two antigens; the third technique is similar to the previous one, but we have additionally inserted the FMDV 2A gene in frame with those of the antigens; and the last approach consists of the cloning of a multicistronic plasmid which expresses a cap-dependent first antigen and IRES-dependant second antigen. In all the constructs the first encoded antigen is the rabies virus glycoprotein and the second is the hemagglutinin glycoprotein of the CDV. The expression of the encoded antigens was evidenced by *In vitro* transfection of BHK-21 cells with the different DNA-based vaccine candidates and immunohistochemical identification of the corresponding antigens. The efficiency of multivalent DNA-based vaccination was evaluated by inoculating mice or dogs with the corresponding plasmids and subsequently assaying the induced virus-neutralizing-antibodies against both valences. Against the rabies virus and the CDV, neutralizing antibodies highly correlate with the induced protective effects.

## Methods

### Viruses, cells and commercial vaccines

Pasteur Virus strain (PV) for the rabies valence and the Onderstepoort (OP-CDV) strain for the CDV valence were used for virus-neutralizing antibody assays. VERO cells were used for the production of OP-CDV and for antibody seroneutralization assays against CDV. BHK-21 cells were used for the propagation of rabies PV strain, for antibody assays using the WHO reference technique RFFIT (Rapid Focus Fluorescent Inhibition Test) and for *in vitro* expression of the different candidate DNA-based vaccines. Rabisin® (Merial, France), is an adjuvanted and inactivated vaccine against rabies, prepared from the rabies virus multiplied in NIL2 cells (established in line at the Wistar Institute Philadelphia, USA, from a culture of hamster embryo cells). Tetradog® (Merial, France), is a vaccine against the major canine diseases (canine distemper, adenoviroses, parvovirosis, and *L. canicola* and *L. icterohaemorragiae* leptospiroses).

### Plasmids

**Figure 1:**
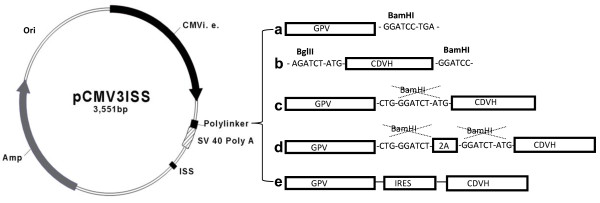
**Schematic representation of monovalent and multivalent plasmids used for DNA-based vaccination.** The presented inserts are for the constructs: **a-** pCMV3ISS-GPV; **b-** pCMV3ISS-CDVH; **c-** pCMV3ISS-GPV-CDVH; **d-** pCMV3ISS-GPV-2A-CDVH; and **e-**. pCMV3ISS-GPV-IRES-CDVH.

The plasmid backbone pCMV3ISS and pCMV3ISS-GPV (Figure 1a) encoding to the PV strain glycoprotein (GPV) of the rabies virus were constructed as described [12].

In order to construct the CDV DNA-based vaccine candidate, the hemagglutinin glycoprotein gene (CDVH) was amplified by RT-PCR using the viral RNA of the Onderstepoort-CDV strain as a matrix and the following set of primers:

CDVH-BglIIup : 5^′^-AAA GAT CTA TGC TCT CCT ACC AAA GAC AAG-3^′^

CDVH-BamHIrev: 5^′^-AAG GAT CCT CAG GGA TTT GAA CGG TTA C-3^′^

PCR product of CDVH was inserted into the plasmid pCMV3ISS after its linearization with the restriction endonuclease SmaI in order to obtain the candidate DNA-based vaccine pCMV3ISS-CDVH (Figure 1b).

The construction of pCMV3ISS-GPV-CDVH, which encodes to a fusion poly-protein of GPV and CDVH, was obtained after the insertion of CDVH gene extracted from pCMV3ISS-CDVH by digestion with BglII and BamHI to pCMV3ISS-GPV after linearization with BamHI (Figure 1c).

For the construction of the candidate DNA-based vaccine pCMV3ISS-GPV-2A-CDVH, which encodes to a fusion poly-protein including the GPV and CDVH with the FMDV 2A oligopeptide in between, we have started with the annealing of the double-stranded DNA of the 2A insert. Hence, we have designed the following set of complimentary primers:

2Aup: 5^′^-GAT CTA ATT TTG ACC TTC TCA AGT TGG CGG GAG ACG TCG AGT CCA ACC CTG GGC CC-3^′^

2Arev: 5^′^-GAT CCG GGC CCA GGG TTG GAC TCG ACG TCT CCC GCC AAC TTG AGA AGG TCA AAA TT-3^′^

The primers were mixed in the same Eppendorf tube, denatured for 5 minutes in boiling water and finally annealed for 30 min at 60°C in a water bath. The resulting double stranded DNA fragment has 5^′^ BglII and 3^′^ BamHI compatible ends. This DNA fragment was inserted to pCMV3ISS-GPV after its linearization with BamHI to give pCMV3ISS-GPV-2A with a unique BamHI site downstream to the 2A gene. This construct was digested by BamHI and inserted the CDVH gene after its extraction from pCMV3ISS-CDVH digestion with BglII and BamHI, to result in pCMV3ISS-GPV-2A-CDVH (Figure 1d).

The construction of pCMV3ISS-GPV-IRES-CDVH, which is a bicistronic plasmid encoding to a cap-dependant GPV and to an IRES-dependant CDVH, was conducted in different steps. The IRES sequence was amplified by PCR using the plasmid pUMVC3-mIL12 (Aldevron, Street South-Fargo, ND) as a matrix and the following set of primers:

IRES-BglIIup : 5^′^-AAA GAT CTT AAT TCC GCC CCT CTC CCC-3^′^

IRES-BamHIrev : 5^′^-GGA TCC ATT ATC GTG TTT TTC AAA GG-3^′^

Then, the PCR product was digested by BglII and BamHI restriction endonucleases and inserted to pCMV3ISS-GPV after linearization with BamHI, which is upstream prior to the stop codon of the rabies glycoprotein. The resulting plasmid (pCMV3ISS-GPV-IRES) was digested with BamHI and inserted with a CDVH gene extracted after the digestion of pCMV3ISS-CDVH with BglII and BamHI, and resulted in pCMV3ISS-GPV-IRES-CDVH (Figure 1e).

For plasmid purification, the Qiagen kit (Qiagen Gmbh, Germany) was used according to manufacturer recommendations.

### *In vitro* transfection and immunofluorescent detection

For *in vitro* transfection in BHK-21 cells of the different candidate DNA-based vaccines, the “SuperFect Transfection Reagent” (Qiagen Gmbh, Germany) was used according to the manufacturer’s instructions. Briefly, 9,000 BHK-21 cells per well were incubated at 37°C overnight in a 96-well cell culture plate. A mixture of plasmid and the superfect reagent was incubated for 3 hours with the cells. After washing and addition of 200 μl of DMEM with 10% fetal calf serum, the cells were incubated for 48 hours before the revealing of the expressed proteins.

The revealing was carried out by following standard techniques. Briefly, the cells were washed and fixed, then incubated at 37°C for 1 h with either an anti-rabies glycoprotein monoclonal antibody produced in mice (HyTest Ltd, Turku, Finland), or with an anti-CDV polyclonal antibody produced in dogs. After washing, the cells were further incubated at 37°C for 1 h with either a mouse or dog anti-IgG, both FITC conjugated. Finally, the cells were washed and observed under a fluorescent microscope (original magnification × 400).

### Inoculation of mice

BALB/c mice (IFFA credo, France) were bred in the “Institut Pasteur de Tunis” animal facilities. Female mice between 6 and 8 weeks old were used and were treated according to local regulations and by a properly qualified personnel. Each group of mice was composed of 5 animals. Before inoculation, the mice were anaesthetized with 1.8 mg of sodium pentobarbital in 200 μl of physiological water, intraperitoneally. Mice were immunized with 50 μg of Qiagen purified plasmid of the corresponding DNA vaccine candidate injected intramuscularly in 100 μl of PBS, 50 μl in each anterior tibialis muscle. Sham vaccination consisted of a single injection of 50 μg of Qiagen purified plasmid backbone pCMV3ISS in the same conditions as above. Non-vaccinated control mice received no treatment at all (PBS group). Experimental research on mice has been performed with the approval of the National Ethics Committee “Comité d’Ethique de l’Institut Pasteur de Tunis”, with the reference number “IPT/LMVBD/13/2012).

### Inoculation of dogs

Two-month-old puppies from the common local mongrel breed were reared in a kennel in experimental conditions for 4 months. They were initially deparasitized, properly fed and at 6 months of age they were inoculated the different vaccine preparations. Throughout the experiments the animals were treated according to local regulations and by properly qualified personnel. Three groups of dogs were composed. The 4 dogs in the first group were inoculated intradermally with 100 μg of the plasmid pCMV3ISS-CDVH, by using the “Dermo-Jet Injector” (AKRA, France), with two shots in the inner face of each ear. The 4 dogs in the second group were administered subcutaneously the commercial vaccine Tetradog®. The remaining 3 dogs in the third group were administered 100 μg of the plasmid backbone pCMV3ISS in the same conditions as with pCMV3ISS-CDVH. Blood samplings were carried out at days 0, 30, 60, 90 and 365 post-vaccination. The sera were collected and conserved at −20°C until use for CDV-neutralizing antibody assays. Experimental research on dogs has been performed with the approval of the National Ethics Committee “Comité d’Ethique de l’Institut Pasteur de Tunis”, with the reference number “IPT/LMVBD/13/2012).

### Antibody assays

Assay of rabies-virus-neutralizing antibodies was carried out by using the RFFIT technique as reported [23]. For the assay of CDV-neutralizing antibodies, serial dilutions of either mouse or dog sera in Dulbecco’s Minimal Essential Medium (DMEM), supplemented with 10% fetal calf serum, were prepared in a sterile 96-well tissue culture plate. Fifty μl of CDV-Onderstepoort strain (100 plaque-forming units per well) were added to each well (excluding cell controls), and plates were incubated for 1 h at 37°C. One hundred μl of a suspension containing 2.10^5^ Vero cells were added to each well and the plates were incubated for 4 to 6 days at 37°C. Titers were expressed as the highest dilution of sera that inhibited the cytopathic effect of CDV-Onderstepoort strain.

### Statistical analysis

EpiCalc 2000 version 1.02 was used for statistical analysis. To compare two means, a t-statistic test is used; given the means, standard deviations and sample sizes as determined by the Microsoft Excel Package. For rates comparisons, two by two tables were used. Rates and sample sizes are needed for the t-statistic tests. To calculate the p-value of the t-statistic test, the method uses the series summation. For means and rates comparisons, p-values lower than 0.05 are significant at the 95% confidence interval and higher than that are not significant.

## Results

### *In vitro* expression of monovalent and multivalent DNA-based vaccine candidates

**Figure 2:**
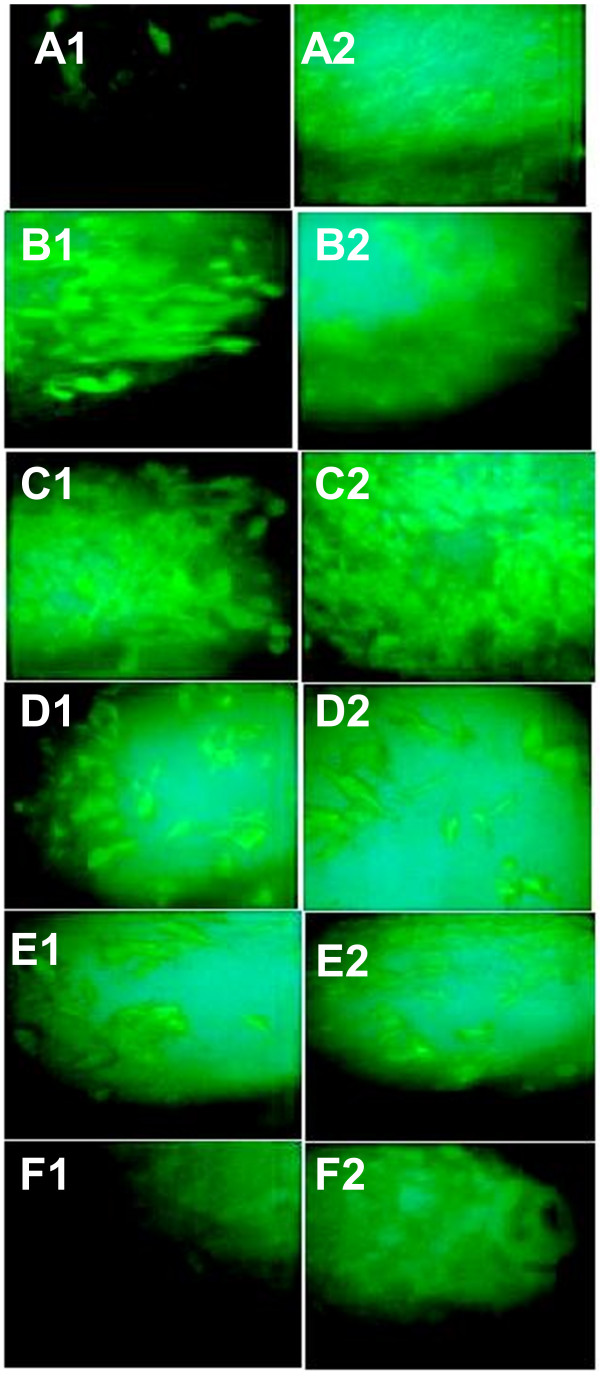
**Cell transfection with the different DNA-based vaccine candidates.** Cells were transfected with pCMV3ISS-GPV (**A1**); pCMV3ISS-CDVH (**A2**); pCMV3ISS-GPV and pCMV3ISS-CDVH (**B1** and **2**); pCMV3ISS-GPV-CDVH (**C1** and **2**); pCMV3ISS-GPV-2A-CDVH (**D1** and **2**); pCMV3ISS-GPV-IRES-CDVH (**E1** and **2**); and pCMV3ISS (**F1** and **2**). **A**-**F1**, were first incubated with a murine anti-rabies glycoprotein monoclonal antibody, then with anti-IgG of mouse conjugated with FITC. **A**-**F2** incubated with anti-CDV polyclonal antibodies produced in dogs, then with anti-IgG of dog FITC conjugated. Cells were observed under a fluorescent microscope (original magnification × 400).

We have transfected BHK-21 cells with the different monovalent and multivalent plasmids and revealed the expression of the encoded antigens. For the monovalent plasmids, almost all the cells in the wells of the culture plates have expressed the encoded proteins (Figure 2: A1 for pCMV3ISS-GPV and A2 for pCMV3ISS-CDVH). When the transfections were with the multivalent plasmids, which encode to GPV and CDVH at the same time, BHK-21 cells have expressed both antigens evenly. Furthermore, similar to the monovalent plasmids, almost all the cells in the corresponding wells expressed both recombinant proteins. Hence, efficient *in vitro* expression of the encoded antigens was obtained after the use of a cocktail of plasmids (Figure 2 B1 and B2), or a plasmid which encodes to a fusion poly-protein with or without the FMDV 2A oligopeptide (Figure 2 C1 and C2, D1 and D2, respectively), or a bicistronic plasmid (Figure 2 E1 and E2).

### Immune responses of the monovalent DNA-based vaccine candidate against CDV in mice and dogs

**Figure 3:**
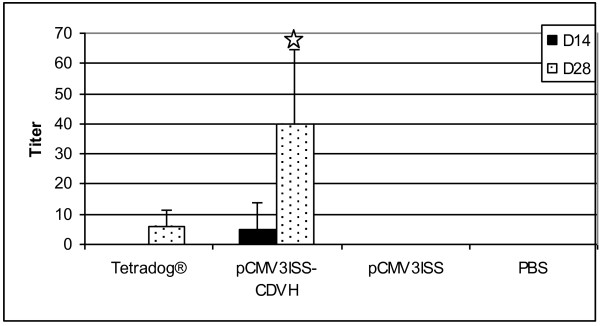
**CDV-neutralizing antibody titers in mice inoculated with pCMV3ISS-CDVH or Tetradog®.** Groups of mice were administered intramuscularly either 50 μg of pCMV3ISS-CDVH; or 200 μl of Tetradog® and compared to sham vaccinated mice (50 μg of pCMV3ISS) and non-vaccinated mice (PBS). All the mice were blood-sampled two and four week’s post-vaccination. Titers were expressed as the highest dilution of sera that inhibited the cytopathic effect of CDV-Onderstepoort strain. Results correspond to the mean antibody titers (the highest dilution of sera that inhibited the cytopathic effect of CDV-Onderstepoort strain) of 5 mice per group. Stars indicate statistical significances of differences between groups of mice administered either pCMV3ISS-CDVH, or Tetradog®.

We have already shown the efficiency of DNA-based vaccination against rabies in the mouse and dog models [12,13]. Here we have tested the monovalent DNA-based vaccine candidate pCMV3ISS-CDVH which encodes to the hemagglutinin glycoprotein of the canine distemper virus. We have tested the capacity of this construct in inducing humoral immune responses in BALB/c mice and compared them to those generated by cell culture based vaccination. As shown by Figure 3, at day 14 post-vaccination, none of the mice inoculated with cell culture based vaccine (Tetradog®) has developed a detectable level of virus-neutralizing antibodies. However, out of 5 mice inoculated with pCMV3ISS-CDVH, 2 of them produced specific neutralizing antibodies higher or equal to the threshold of 10; the mean in the group being 5. Two weeks later, in the group Tetradog®, 3 mice out of 5 had exactly the titers of 10; and in the remaining 2, the antibodies remained undetectable (the mean was 6). Comparatively, the mean for mice inoculated with DNA was higher 40 (p-value 0.017). Furthermore, all the mice in the group developed antibody titers greater than or equal to 20.

**Figure 4:**
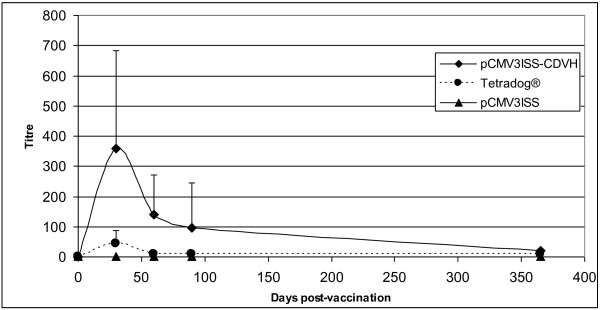
**Kinetics of CDV-neutralizing antibody titers in dogs inoculated pCMV3ISS-CDVH or Tetradog®.** Groups of 4 dogs were administered either 100 μg of pCMV3ISS-CDVH, intradermally, or 1 ml of Tetradog®, intramuscularly and compared to a group of 3 sham-vaccinated animals (100 μg of pCMV3ISS). All the dogs were blood-sampled at days 0, 30, 60, 90 and 365 post-vaccination. Titers were expressed as the highest dilution of sera that inhibited the cytopathic effect of CDV-Onderstepoort strain. Results correspond to the mean antibody titers (the highest dilution of sera that inhibited the cytopathic effect of CDV-Onderstepoort strain) of the corresponding dogs in each group.

The humoral immune potency of the same construct was evaluated in dogs. As illustrated by Figure 4, at one month post-vaccination, the titers in inoculated dogs with pCMV3ISS-CDVH oscillated between 80 and 640 with a mean of 360. At 60 and 90 days post-vaccination, the mean titers dropped to 140 and 95, respectively. At one year post-vaccination, each dog maintained a titer of 20. Therefore, throughout the follow up, the dogs raised antibody titers superior to the threshold of 10, which is considered as being protective against a CDV infection. Tetradog® subcutaneously inoculated animals showed lower neutralizing antibody responses compared to those inoculated pCMV3ISS-CDVH. At one month post-vaccination, 1 out of the 4 dogs did not seroconvert and maintained that status during the entire observation period. During the same period, the remaining 3 dogs raised titers varying between 20 and 80 (mean 45). The mean titers dropped to 10 at day 60, then to 7.5 at days 95 and 365 post-vaccination.

We can conclude that in both mice and dogs, similar to what we have reported with rabies, DNA-based vaccination against CDV is more capable of inducing specific neutralizing antibodies than cell-culture-derived vaccines.

### Anti-rabies immune responses of multivalent DNA-based vaccine candidates in mice

*In vitro* expression of the different multivalent DNA-based vaccine candidates have shown that all the adopted strategies are capable of efficiently expressing both encoded antigens. The purpose of such constructs is a rather efficient *in vivo* expression of the encoded antigens and the induction of strong immune responses. With rabies and CDV, the correlate of protection and the strength of the induced immune responses are represented by the level of production of virus-neutralizing antibodies. We have inoculated different groups of BALB/c mice with the different monovalent or multivalent plasmids and assayed in a first step their capacities to induce rabies-virus-neutralizing antibodies. The induced antibody responses were compared between the different groups of mice in order to choose the more appropriate strategy to design a multivalent plasmid. In addition, we have compared the humoral immune responses when mice were vaccinated with either monovalent or multivalent DNA-based vaccine candidates, or when they were vaccinated with classical cell culture based vaccines (Rabisin®).

**Figure 5:**
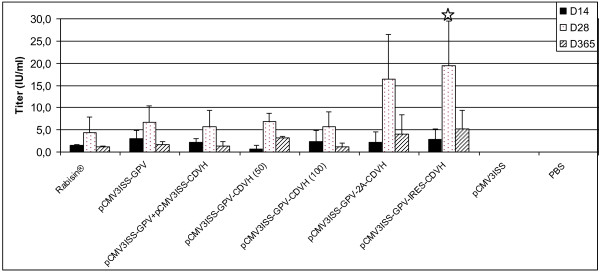
**Rabies-virus-neutralizing antibody titers in mice inoculated with different monovalent or multivalent DNA-based vaccine candidates or cell-culture-derived vaccines.** Groups of mice were administered intramuscularly either pCMV3ISS-GPV; or pCMV3ISS-GPV-CDVH; or pCMV3ISS-GPV-2A-CDVH; or pCMV3ISS-GPV-IRES-CDVH, 50 μg each, or pCMV3ISS-GPV and pCMV3ISS-CDVH (50 μg, each); or pCMV3ISS-GPV-CDVH (100 μg); or 200 μl of Rabisin® and compared to sham vaccinated group (50 μg of pCMV3ISS) and non-vaccinated group (PBS). All the mice were blood-sampled at Days 14, 28 and 365 post-vaccination. Results correspond to mean antibody titers of 5 mice per group expressed in IU/ml of serum as measured by RFFIT. Stars indicate statistical significances of differences between groups of mice administered the multivalent plasmid compared to those administered the monovalent plasmid pCMV3ISS-GPV.

As shown by Figure 5, mice vaccinated with a single dose of Rabisin® developed a low level of virus-neutralizing antibodies at day 14 (1.5 IU/ml), which peaked at day 28 (4.4 IU/ml), then dropped to 1.1 IU/ml 11 months later. When mice were inoculated with the monovalent plasmid pCMV3ISS-GPV, slightly higher, but statistically not significant, antibody responses were obtained with a peak of 6.7 IU/ml at day 28 (p-value 0.348). When the same plasmid was inoculated in mice in association with the plasmid pCMV3ISS-CDVH, no substantial changes of the induced responses were obtained, with a peak of 5.7 IU/ml (p-value 0.68). Therefore, we can consider that the use of a cocktail of plasmids has neither beneficial, nor deleterious effects in the capacity of inducing rabies-virus-neutralizing-antibodies. The administration of either 50 μg or 100 μg of the multivalent plasmid pCMV3ISS-GPV-CDVH induced slightly variable humoral immune responses. The inoculation of the higher quantity of plasmid seems to induce a higher response at day 14 (2.4 IU/ml compared to 0.7, p-value 0.1669) and very close peaks at day 24 (6.9 IU/ml compared to 5.8, p-value 0.522), although in both cases the differences are statistically not significant. For mice inoculated with either the plasmid pCMV3ISS-GPV-2A-CDVH or pCMV3ISS-GPV-IRES-CDVH, the onset of the immune responses were identical to those administered with the monovalent plasmid (2.2 IU/ml and 2.8 IU/ml, respectively). Later on, for both multivalent constructs, the induced rabies-virus-neutralizing antibodies are higher than those of the monovalent pCMV3ISS-GPV. Therefore, mice inoculated with the plasmid which generates GPV and CDVH after the cleavage of the FMDV 2A oligopeptide, reached the titer of 16.4 IU/ml (p-value 0.068) at day 28, which then dropped to 4.1 IU/ml (p-value 0.25) at day 365. For mice inoculated with the bicistronic plasmid pCMV3ISS-GPV-IRES-CDVH, the mean titer was 19.4 IU/ml (p-value 0.029) at day 28, which then dropped to 5.2 IU/ml (p-value 0.10) 11 months later.

### Anti-CDV immune responses of multivalent DNA-based vaccine candidates in mice

**Figure 6:**
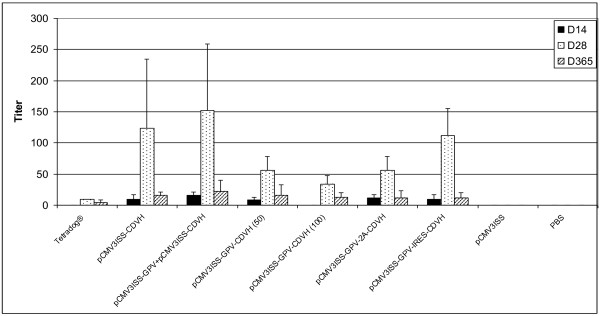
**CDV-neutralizing antibody titers in mice inoculated with different monovalent or multivalent DNA-based vaccine candidates or cell-culture-derived vaccines.** Groups of mice were administered intramuscularly either pCMV3ISS-CDVH; or pCMV3ISS-GPV-CDVH; or pCMV3ISS-GPV-2A-CDVH; or pCMV3ISS-GPV-IRES-CDVH, 50 μg each, or pCMV3ISS-GPV and pCMV3ISS-CDVH (50 μg, each); or pCMV3ISS-GPV-CDVH (100 μg); or 200 μl of Tetradog® and compared to sham-vaccinated group (50 μg of pCMV3ISS) and non-vaccinated group (PBS). All the mice were blood-sampled at Days 14, 28 and 365 post-vaccination. Titers were expressed as the highest dilution of sera that inhibited the cytopathic effect of CDV-Onderstepoort strain. Results correspond to the mean antibody titers (the highest dilution of sera that inhibited the cytopathic effect of CDV-Onderstepoort strain) of 5 mice per group.

The same mice vaccinated with the multivalent plasmids were assayed for their abilities to induce CDV-neutralizing antibodies and compared to those vaccinated with either Tetradog® or the monovalent plasmid pCMV3ISS-CDVH (Figure 6). At day 14 post-vaccination, neither of the mice vaccinated with Tetradog® mounted any detectable level of virus-neutralizing antibodies. At day 28, each mouse has a titer of 10 with the mean dropping to 4 at one year post-vaccination. Hence, cell-culture-derived anti-CDV vaccine in mice was only capable of inducing low levels of antibody responses. Comparatively, mice inoculated with the monovalent plasmid, which encodes to CDVH, mounted a humoral immune response (10 for each mice) at day 14 and reached a peak of 124 (p-value 0.049) two weeks later, which then dropped to 16 (p-value 0.0046) at day 365. When the same plasmid was injected as a cocktail with pCMV3ISS-GPV, at least the same titers were reached post-vaccination: 16 (day 14), 152 (day 28) and 22 (day 365). Hence, the rabies valence did not show any humoral immune inhibition effect towards the CDVH valence, if not boosting it. The plasmid which encodes to the fusion poly-protein (GPV-CDVH) seems to induce slower and lower onsets of humoral immune responses compared to the use of the monovalent plasmid, especially at day 28 when the inoculation was with 100 μg of plasmid (34 p-value 0.107), but the difference is statistically not significant. The plasmid which encodes to the fusion of GPV-2A-CDVH showed that its capacity to induce CDV-neutralizing antibodies was unchanged regardless of whether the 2A oligopeptide was included in the construct. Hence, mice inoculated with the same quantity of plasmid of either pCMV3ISS-GPV-CDVH or pCMV3ISS-GPV-2A-CDVH, generated the same trends of humoral immune responses. The most efficient multivalent plasmid in inducing CDV-neutralizing antibodies was the bicistronic pCMV3ISS-GPV-IRES-CDVH. It was able to onset the humoral immune responses identically to the monovalent plasmid. Furthermore, at day 28 post-vaccination, almost the same high level reached by mice inoculated with pCMV3ISS-CDVH was obtained after the inoculation of the multicistronic plasmid. Hence, even though the expression of CDVH valence was IRES dependant, the induced humoral immune responses were equivalent to those of the monovalent plasmid.

## Discussion

Rabies is still considered to be an important zoonoses in most of the developing countries and stray dogs represent the main vector and reservoir for this disease. The most efficient way to prevent the transmission of rabies to humans is by vaccinating dogs. In developing countries, the turnover rate of dogs is around 30% each year, mostly replaced by new born puppies [24]. More than 42% of the reported rabid dogs in Thailand are younger than 6 months [8]. During the vaccination campaigns, puppies younger than 3 months are not targeted. Owing to the yearly recurrence of the vaccination campaigns against rabies, unvaccinated puppies during the campaign keep that status for at least one year of their early lives. Consequently, we should persuade local populations to vaccinate their new born puppies against rabies. Furthermore, it is well known that ownerless and stray dogs are rarely administered a rabies vaccination. Thus, the vaccination of new born puppies before becoming ownerless and stray dogs allows residual rabies immunity; otherwise this will never be achieved. Unfortunately, it was well shown that during mass vaccination of dogs against rabies, a big proportion of the vaccinated puppies will die because they were highly exposed to infections with either canine parvovirus or distemper virus because of the intervention of the vaccinators. For this reason, puppy owners are reluctant to vaccinate them against rabies. To overcome this problem, we think it is necessary to include other valences in the inoculated vaccine against rabies, especially against CPV and CDV. We have already shown the efficiency of DNA-based vaccination against rabies in the mouse and dog models [12,13]. Here we have further validated a monovalent DNA-based vaccine candidate (pCMV3ISS-CDVH) which encodes to the hemagglutinin glycoprotein of the canine distemper virus in the same animal models. Hence, multivalent DNA-based vaccination in dogs, including rabies and distemper valences, could represent a strong argument in raising vaccine coverage and efficacy.

We have designed different strategies of multivalent DNA-based vaccination and compared their efficiencies. The use of a cocktail of two plasmids, the first encoding to the rabies glycoprotein, and the second to the hemagglutinin glycoprotein of the CDV, allowed *in vitro* expression and induction in mice of specific virus-neutralizing antibodies against both valences. The induced humoral immune responses were identical to what was obtained when the inoculation was a single monovalent plasmid. This strategy associating two plasmids in the vaccine preparations has bioprocessing drawbacks, since there is a need for two separate processes, quality controls for each plasmid preparation, and mixing them.

The strategy of using a plasmid, which encodes to a fusion polyprotein without an internal cleavage element, induced in mice the same level of neutralizing rabies virus antibodies as the monovalent plasmid (pCMV3ISS-GPV), whether it was administered alone or in association with the plasmid pCMV3ISS-CDVH. Against the CDV valence, the induced humoral immune responses were rather depressed by the fusion of the two valences. In both cases (against rabies and CDV), and whether 50 μg or 100 μg of the plasmid pCMV3ISS-GPV-CDVH were inoculated to mice, the induced humoral immune responses did not change significantly. The introduction of the FMDV 2A gene between those of both antigens did not significantly improve the humoral responses against CDV. However, it seems that when the GPV is *in vivo* liberated from the fusion polyprotein, there is a boost of the immune responses against the rabies virus compared to the use of the monovalent plasmid. Hence, it can be postulated that when the CDVH native valence was expressed in the same cell as GPV, it was able to induce a cross boost effect. Similar results have been reported when a mixture of antigens was used [25,26]. This can be explained by the induction of a more robust cytokine environment since it is reported that DNA-based vaccination is highly efficient in inducing T cell responses [27].

The last strategy based on IRES multicistronic plasmid seems to be the most efficient in inducing rabies virus and CDV-neutralizing antibodies. The beneficial cross effect of CDVH on rabies valence was at least the same with pCMV3ISS-GPV-IRES-CDVH as with pCMV3ISS-GPV-2A-CDVH. In addition, against CDV, mice inoculated with the bicistronic plasmid raised the same level of antibody response as those administered the monovalent plasmid. Compared to mice inoculated with the cocktail of the two monovalent plasmids (50 μg of each plasmid), those inoculated with the bicistronic plasmid gave better immune responses with a total quantity of 50 μg. Furthermore, it has been reported that a gene inserted upstream from the IRES is strongly cap-dependent expressed, while a gene placed downstream is IRES-dependent expressed at a lower level [16,17]. Nevertheless, in our construction, even if there is a down expression of CDVH after the inoculation of mice with the bicistronic plasmid, the induced humoral immune responses were similar to that after the administration of the monovalent pCMV3ISS-CDVH plasmid. Therefore, the IRES strategy is not only beneficial at the immunogenecity level, but also at the bioprocessiong one. This multivalent approach is based on a single construct, allowing for a more straightforward processing and quality control. The cost of production is further restrained since only the half quantity of plasmid is needed for at least the same level of immune responses.

If we compare any of the DNA-based multivalent vaccine candidates with the use of cell culture vaccines, it is quite clear that they are more efficient, especially against the CDVH valence. Against the rabies valence, we have already reported that DNA-based vaccination is superior to the inoculation of commercial cell culture vaccines [21,23]. With pCMV3ISS-GPV-IRES-CDVH, the induced immune responses are further improved compared to those induced with Tetradog® (p-value 0.0008, at day 28 post-vaccination) and Rabisin® (p-value 0.013, at day 28 post-vaccination).

## Conclusions

We have designed different multivalent plasmids and we have shown that the IRES based bicistronic construct is the most efficient. It did not only induce better humoral immune responses in mice compared to those administered cell-culture-derived vaccines, but also better than after the use of monovalent plasmids. The use of FMDV 2A, inserted between the genes of two antigens, was also efficient for the design of multivalent DNA vaccine candidates. Such plasmids will be very beneficial for DNA-based vaccination against rabies in dogs. This approach will allow the targeting in a single shot, not only against rabies, but also against other deadly diseases of puppies, such as canine distemper. Hence, it will efficiently contribute to the increase of the vaccination coverage against rabies by persuading dog owners to include their puppies during the national campaigns. Nevertheless, more work is necessary to develop this kind of vaccine for final use, in addition to including a CPV valence.

## Competing interests

The authors declare that they have no competing interests.

## Authors’ contributions

LT constructed all vectors described in this manuscript and performed all *in vitro* and animal experimentations and the immunological investigations and has been involved in drafting the manuscript. SBA, YC and RD have participated in animal experimentation. CB contributed by designing and supervising the work, drafting the manuscript, and carrying out the statistical analysis. All authors read and approved the final manuscript.
